# Supplementary material for: Single-Side Superhydrophobicity in Si3N4-Doped and SiO2-Treated Polypropylene Nonwoven Webs with Antibacterial Activity
Source: Polymers (Basel). 2022 Jul 21;14(14):2952. doi: 10.3390/polym14142952 (PMC9323961; doi:10.3390/polym14142952)
Supplement: Supplementary file 1 [file polymers-14-02952-s001.zip › polymers-1807116-supplementary.pdf]

Single-side superhydrophobicity in Si_3N_4-doped and SiO_2-treated polypropylene nonwoven webs with antibacterial activity and good performances

Ming-Chao Han [1], Shun-Zhong Cai [1], Ji Wang[1], Hong-Wei He [1,2]*

[1] *Shandong Center for Engineered Nonwovens, Industrial Research Institute of Nonwovens & Technical Textiles, College of Textiles & Clothing, Qingdao University, Qingdao 266071, Shandong, China.*

[2] *State Key Laboratory of Bio-Fibers and Eco-Textiles, Qingdao University, Qingdao 266071, Shandong, China*

Correspondence should be addressed to hhwpost@163.com

Melt flow rate measurement

The flow performance of electret masterbatch was tested by a melt flow rate meter (XNR-400a, Jinhe, China). The test method was according to GB/T 3682-2000, the test temperature was 230°C and the nominal load was 2.16kg.

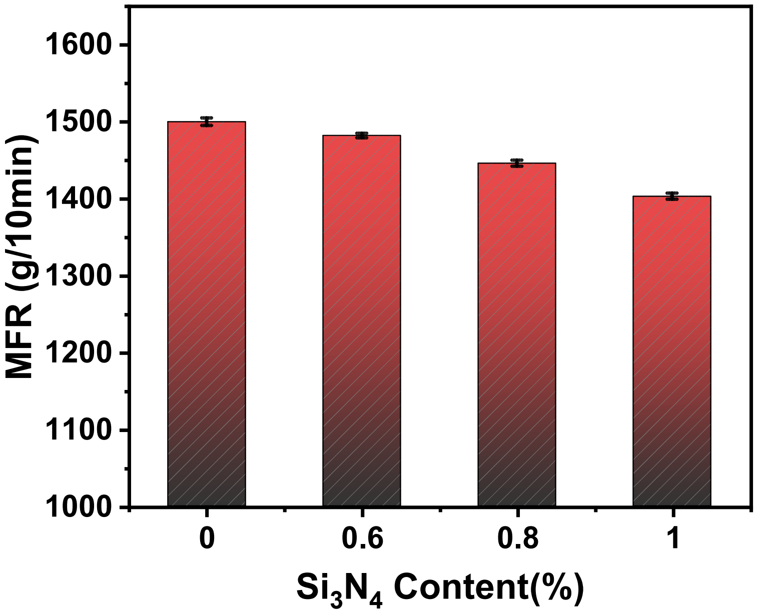

Figure.S1 Flow rates of masterbatch melt with different Si_3N_4 contents

As shown in Figure.S1, with the increase of silicon nitride content, the flowability of PP becomes worse, decreasing from 1500 g/1000 min to 1403 g/10 min. The addition of silicon nitride increases the viscosity of the PP system, and the flow performance deteriorates.
